# The primary systemic vasculitis associated optic neuritis: a retrospective analysis in a single center over 10 years

**DOI:** 10.1007/s10792-024-03307-2

**Published:** 2024-09-23

**Authors:** Simeng Tang, Hang Zhou, Rui Li, Yu Wang, Hongyang Li, Yanli Hou

**Affiliations:** 1 Department of Ophthalmology, Beijing Friendship Hospital, Capital Medical University, Beijing, 100050 China; 2 Department of Rheumatology, Beijing Friendship Hospital, Capital Medical University, Beijing, 100050 China; 3 Department of Radiology, Beijing Friendship Hospital, Capital Medical University, Beijing, 100050 China; 4 Department of Nuclear Medicine, Beijing Friendship Hospital, Capital Medical University, Beijing, 100050 China

**Keywords:** Optic neuritis, Primary systemic vasculitis, Takayasu’s arteritis, Giant cell arteritis, Antineutrophil cytoplasmic antibody-associated vasculitis

## Abstract

**Objectives:**

To investigate the clinical and image characteristics of primary systemic vasculitis-associated optic neuritis patients.

**Methods:**

This is a retrospective study. The patients clinically diagnosed with primary system vasculitis-induced optic neuritis were recruited from March 2013 to December 2023. All cases received orbital magnetic resonance imaging scans were analyzed. The ocular findings, systemic manifestations, laboratory data and prognosis were reviewed retrospectively. In addition, the related literature was reviewed.

**Results:**

Fourteen patients (21 eyes), including 10 men and 4 women, were enrolled in this study. The ages ranged from 30 to 86 years in this cohort. Orbits MRI detects the enlargement and/or enhancement of the optic nerve. Cases 1–5 reported a confirmed diagnosis of Takayasu’s arteritis, and cases 6–8 had giant cell arteritis. Cases 9–13 were antineutrophil cytoplasmic antibody-associated vasculitis. Case 14 was Cogan’s syndrome. Mult organs and tissues, such as the kidneys, heart, paranasal sinuses, meninges, and respiratory system, were involved. In all of the 14 involved patients, the disease onset was either during the fall or winter season. There were no or only slight improvements in visual activity after conventional therapies.

**Conclusions:**

The autoantibodies’ attack on the optic nerve, ischemic damage, or destruction of the blood–brain barrier may be the potential pathogenesis of vasculitis-associated optic neuritis. Even with prompt and aggressive clinical interventions, the prognosis remains unsatisfactory.

## Introduction

Primary systemic vasculitis (PSV) involves a heterogeneous group of uncommon pathologies characterized by vesicular inflammation and necrosis of the arteries, veins, and capillary beds in specific tissues or organs. The optic nerve is affected in rare cases but with irreversible visual damage. The most used nomenclature of PSV is according to the size of the affected vessel [1]. Large vessel vasculitis (LVV) presents a necrotizing inflammation involving the large arteries, such as giant cell arteritis (GCA) and Takayasu’s arteritis (TAK). Small vessel vasculitis (SVV) is characterized by a small-vessel necrotizing vasculitis or granuloma formation, which includes antineutrophil cytoplasmic antibody (ANCA)-associated vasculitis (AAV) and immune complex deposition-induced vasculitis. Some patients may have all sizes of vasculitides involved, such as Cogan’s syndrome (CS).

Ocular manifestations are usually detected in 34.1% of granulomatosis with polyangiitis (GPA) patients, 8.9% of microscopic polyangiitis (MPA) patients [2], 10–20% of GCA patients, and < 10% of TAK patients [3]. Notably, an initial diagnosis of ocular vasculitis can be an indicator of upcoming life-threatening PSV conditions. Systemic and local vasculitis occur concurrently or at a later stage of PSV, involving bilaterally or unilaterally eyes [2]. Conjunctivitis, episcleritis, and keratitis are the most common anterior segment manifestations. Orbit involvement includes orbital pseudotumor, nasolacrimal duct obstruction, and proptosis. Vascular complications present as retinal/choroidal artery occlusion [4].

PSV-related optic neuropathies are usually developed due to compression by orbital mass or contiguous spread of inflammation from meninges/orbit/sinuses, or GCA induced- arteritic anterior ischemic optic neuropathy (AAION). Optic neuritis (ON) is exceptionally rare in PSV patients. Both ophthalmologists and rheumatologists paid little attention to PSV-linked optic neuritis (PSV-ON), leading to delays in diagnosis and vision damage. Herein, we present 14 cases (21 eyes) of PSV-ON and review the relevant studies.

## Methods

We retrospectively collected the in-house developed electronic medical records (EMRs) of PSV-ON patients from August 2013 to August 2023 in Beijing Friendship Hospital affiliated with Capital Medical University. The EMRs data contains the patient’s massages, diagnoses, laboratory findings, radiological records, interventions et al. All enrolled cases received ophthalmic assessments before and after the treatment. Visual ability was assessed by the Logarithmic standard visual acuity chart. The orbital and brain magnetic resonance imaging (MRI) examinations were performed before treatment, using a 3.0-T scanner, including T1, T2, and T1 post-gadolinium MRI. All data were collected and analyzed by experienced neuro-ophthalmologists and neuro-radiologists. The diagnosis of PSV was made based on the 2022 American College of Rheumatology/ European League Against Rheumatism (ACR/EULAR) classification criteria [5–8]. All diagnoses were consulted with experienced rheumatologists and nuclear medicine specialists. The diagnosis of PSV-ON was confirmed by evaluating the visual function impairments and characteristic changes of the optic nerve on MRI, like optic nerve hyperintensities in T2-weighted images (T2WI), abnormal enlargement and/or enhancement of optic nerve parenchyma/sheath in T1-weighted postcontrast MRI, and without orbital apex enhancement or inflamed masses compressing the optic nerves. Optic neuritis associated with other neurological diseases was excluded. Such as multiple sclerosis-associated ON and neuromyelitis optica spectrum disorder-associated ON. Optic perineuritis (OPN) and cases without sufficient clinical information were excluded.

This study was approved by the internal ethics committee of the hospital and conducted following the Declaration of Helsinki. All patients provided consent for further analysis and publishing of their medical findings to help the scientific community and doctors make treatment strategies.

## Results

Fourteen cases (21 eyes), including 10 men and 4 women, were enrolled in this study. The ages ranged from 30 to 86 years in this cohort. Cases 1–5 reported a confirmed diagnosis of TAK, and cases 6–8 had GCA. Cases 9–13 were all diagnosed with AAV with multiple organ involvement. Cases 9 and 10 were identified as GPA and positive for cANCA, while cases 10–13 were identified as pANCA-positive MPA for further investigation. Case 14 was diagnosed with CS.

Optic neuritis in 14 patients, 7 cases involved bilateral eyes. Fourteen eyes presented disc edema, and 2 eyes with optic atrophy in 21 affected eyes. Twelve patients manifested retinal/choroidal vasculitis as well as optic neuritis. Such as retinal cotton wool spots, retinal arterial obstruction, vascular wall staining and fluorescein leakage in fluorescein angiography (FA).

The extra-ocular manifestations of the PSV were summarized. The kidneys were involved in six cases (cases 1, 10–14). Case 1 with TAK explores the renal dysfunction induced by renal arteritis. Cases 10–13 with ANCA-arteritis manifest interstitial renal diseases. Four cases (cases 4, 9, 10, 13) had sinusitis and/or mastoiditis. Cases 9 and 11 had hypertrophic pachymeningitis. Myocardial damage occurred in cases 1,3 and 13. Respiratory tract involved in cases 1,10–13. Oculomotor neuritis and ON coexisted in case 3. Acoustic neuritis and ON coexisted in case 14.

**Fig. 1:**
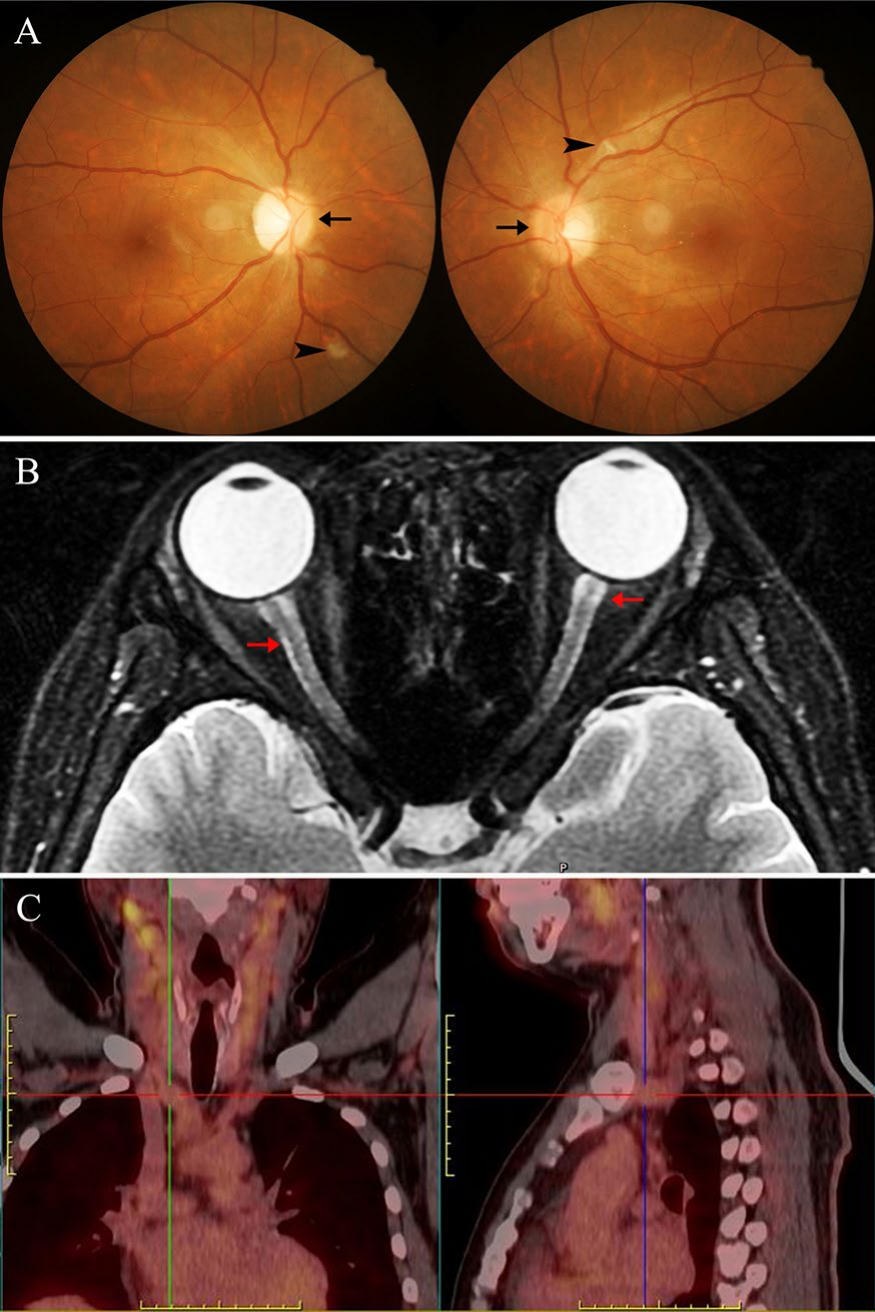
A 32-year-old man. **A**, Fundus photograph: bilateral disc edema (black arrow), retinal cotton wool spots (black arrowhead) presented on both eyes. **B**, Axial T2-weight MRI: a discrete T2-weighed hyperintense signal at both the optic nerve and sneath (red arrow). **C**, 18F-FDG-PET: multivessel disease through high accumulations of radioisotopes (SUVmax:3.7)

**Fig. 2:**
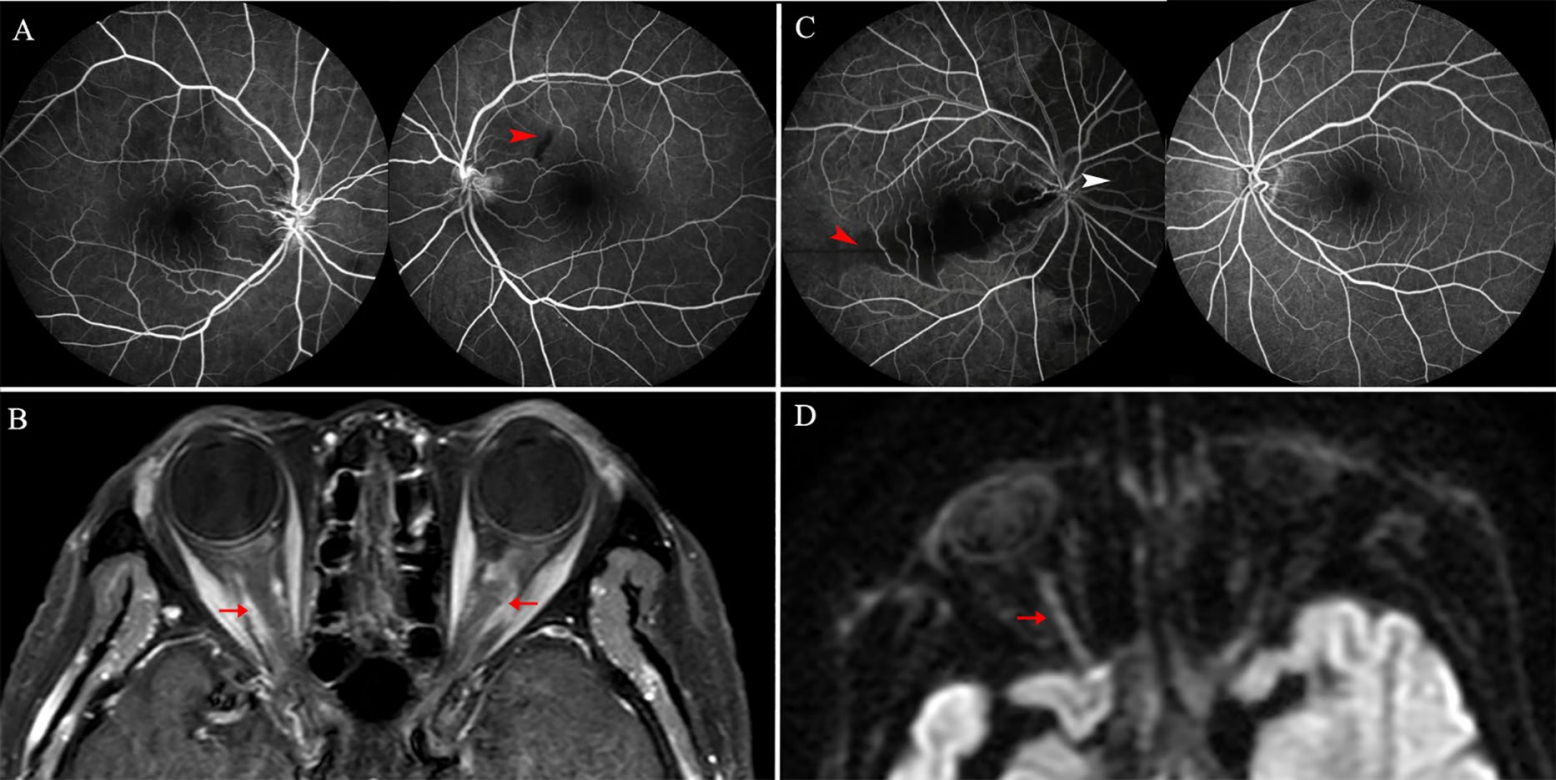
A, B from an 86-year-old woman. **A**, FA: bilateral disc edema with fluorescein leakage. Lesions in the retinal small arteries result in nerve fiber infarcts. (red arrowhead) **B**, Axial T1-weight postcontrast MRI shows enlargement and enhancements of the bilateral optic nerve (red arrow). C, D from a 67-year-old man. **C**, FA: Disc edema, the cilioretinal artery occluded (red arrowhead) and choroidal ischemic area (white arrowhead) presented on the right eye. **D**, DWI: hyperintense DWI signal (red arrow) was seen in the right optic nerve

**Fig. 3:**
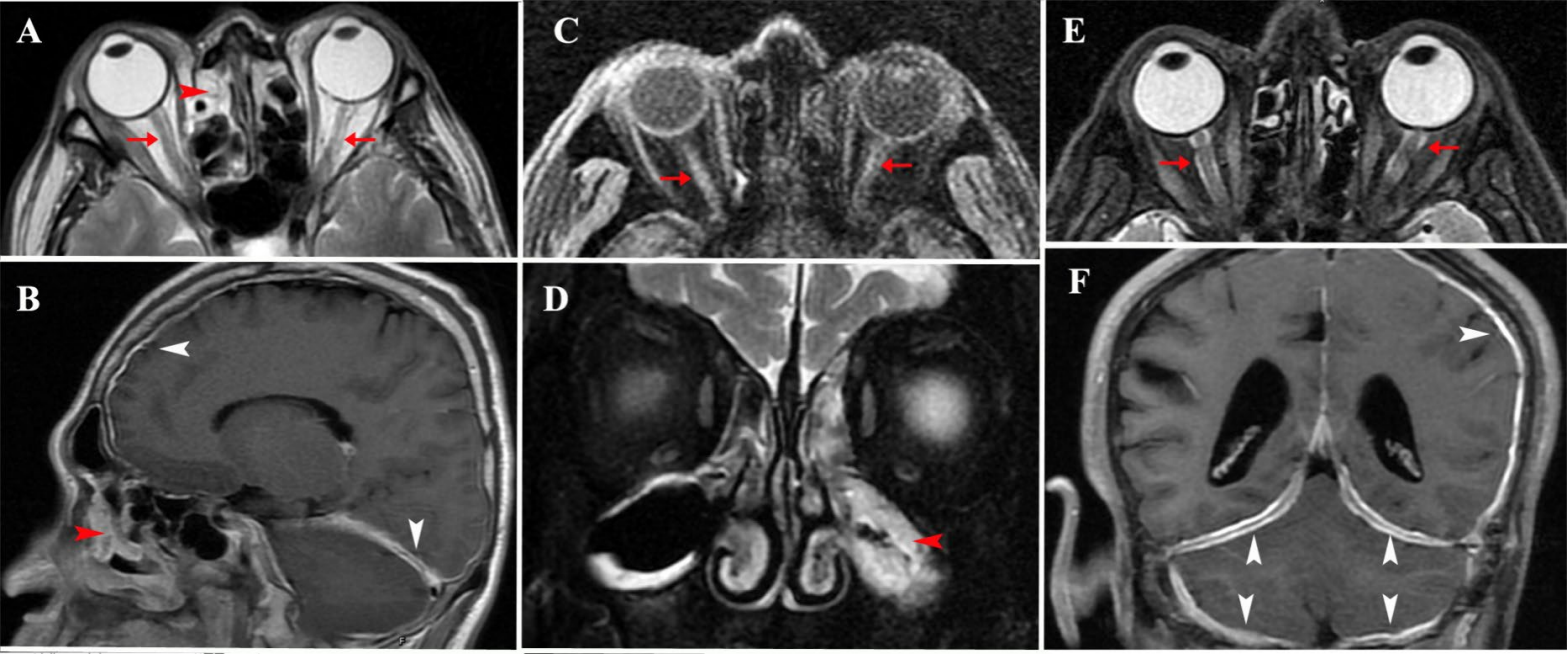
A, B from a 49-year-old man. **A**, axial T2-weighted MRI show hyperintense signals at the bilateral optic nerve (red arrow) and right ethmoid sinus (red arrowhead). **B**: Sagittal T1-weighted postcontrast MRI shows enhancement signals of the maxillary sinus (red arrowhead) and dura mater (white arrowhead). C, D from a 71-year-old man. **C**, axial T1-weighted postcontrast MRI shows enlargement and enhancement of bilateral optic nerves (red arrow). **D** Coronal T2-weighted MRI shows a hyperintense signal at the left maxillary sinus (red arrowhead). E, F from a 58-year-old man. **E**: Axial T2-weighted MRI shows hyperintense signals at bilateral optic nerve and sneath (red arrow). **F**, Coronal T1-weighted postcontrast MRI show the dura mater of bilateral frontal lobes, bilateral tentorium cerebelli parietal, the left parietal, and the left temporal lobes were thickened and enhanced (white arrowhead)

**Fig. 4:**
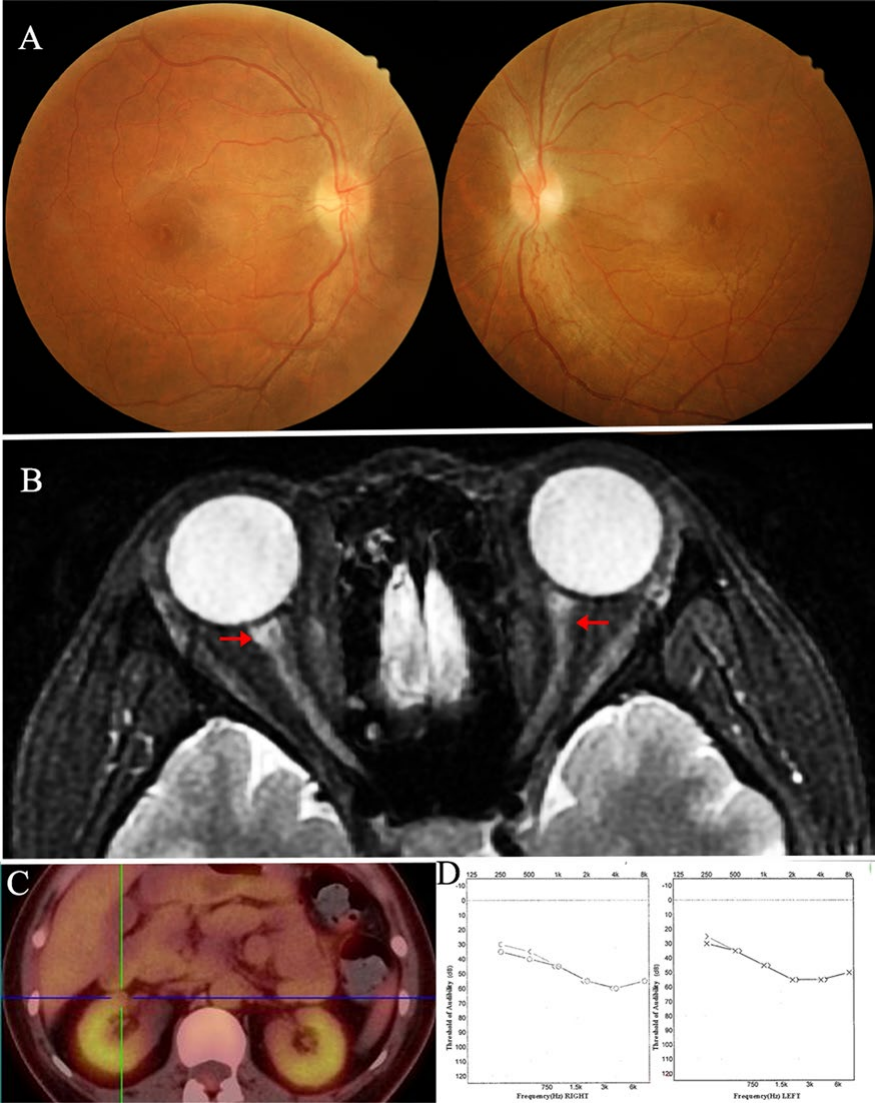
A 30-year-old woman. **A**, Fundus photograph: bilateral optic disc edema, copper-wiring of retinal artery, tortuosity of the central retinal artery. **B**, Axial T2-weighted MRI show hyperintense signal at bilateral optic nerve (red arrow). **C**, 18F-FDG-PET: high accumulation in the renal cortex (SUVmax:5.0). **D**, The threshold of audibility: bilateral moderate to severe sensorineural hearing loss

The extra-ocular image findings of the PSV are present in Figs. 1, 2, 3 and 4. 18F-FDG-PET in Fig. 1 shows high accumulations of radioisotopes in multivessels of TAK patients. Figure 3B shows bilateral ethmoid sinus, maxillary sinus, and dura mater were involved in a 49-year-old man with GPA (case 9). Figure 3D shows discrete T2-weight hyperintense signals at the left maxillary sinus in a 71-year-old man with a GPA (case 10). Figure 3F is a 58-year-old man with MPA (case 11). MRI shows thickened and enhanced signals at the dura mater. Figure 4 is a 30-year-old woman with Cogan’s Syndrome. Her bilateral threshold of audibility shows moderate to severe sensorineural hearing loss. High accumulation in the renal cortex was detected in 18F-FDG-PET.

In all of these cases, the disease onset was either during the fall or winter season. The demographics and clinical data of enrolled cases are summarized in Table 1.

**Table 1:** The demographic and clinical data of enrolled case

Diagnose	Age/Sex		Visual acuity	Visual outcome	Optic disc	Retinal vasculitis	Laboratory findings				Extra-ocular manifestations	Teatments
							CRP (0-8 mg/L)	ESR (0-15 mm/1 h)	MPO-IgG (< 20 U/ml)	PR3-IgG (< 20 U/ml)		
Takayasu’s arteritis												
Case 1	32/M	R	FC	20/200	PE	(+)	259.79	91	(−)	(−)	Renal Dysfunction, Atypical Pathogenspneumonia, Myocardial Damage	3-day i.v 500 mg MP, CCP, Tocilizumab
		L	20/25	20/20	PE							
Case 2	39/M	L	20/32	20/32	Norm	(+)	19.77	57	(−)	(−)	None	9-day i.v 80 mg MP, CCP,MTX
Case 3	30/M	L	20/100	20/50	Norm	(+)	25.9	54	(−)	(−)	Myocardial Damage, Oculomotor neuritis, Anemia	7-day i.v 40 mg MP,MTX,Tocilizumab,IL-6
Case 4	35/M	R	FC	20/200	PE	(+)	8.5	17	(−)	(−)	Antiphospholipid syndrome, Sinusitis	3-day i.v 80 mg MP, MTX
Case 5	65/F	R	FC	FC	OA	(+)	9.07	13	(−)	(−)	None	7-day i.v 40 mg MP, CCP
		L	20/50	20/50	PE							
Giant cell arteritis												
Case 6	86/F	R	FC	FC	PE	(+)	16.75	69	(−)	(−)	Anemia	3-day i.v 240 mg MP
		L	20/25	20/25	PE							
Case 7	67/M	R	HM	NLP	PE	(+)	56.65	50	(−)	(−)	None	3-day i.v 500 mg MP
Case 8	75/M	R	LP	LP	PE	(+)	0.17	NA	(−)	(−)	None	Mecobalamin
ANCA-Arteritis												
Case 9 GPA	49/M	R	20/125	20/125	PE	(+)	53	60	(−)	173.4	Sinusitis,Mastoiditis, Hypertrophic Pachymeningitis	Patient refused
		L	20/20	20/40	PE							
Case 10 GPA	71/M	R	20/32	20/32	Norm	(−)	175	66	149.1	20.5	Interstitial Nephritis, Sinusitis, Mastoiditis, Infection with Bronchiectasis	Oral MP and CCP
		L	20/25	20/25	Norm							
Case 11 MPA	58/M	R	NLP	NLP	OA	(+)	14.93	95	62.4	(−)	Glomerulonephritis, Interstitial Lung Disease Hypertrophic Pachymeningitis	3-day i.v 500 mg MP
		L	20/32	20/32	PE							
Case 12 MPA	58/F	R	20/50	20/50	PE	( −)	32.26	103	110.7	(−)	Renal Dysfunction, Infection with Bronchiectasis, Anemia	Oral MP and CCP
Case 13 MPA	60/M	R	FC	HM	Norm	(+)	70.6	51	106.4	(−)	Renal Dysfunction,Pneumonia. Anemia, Myocardial Damage, Sinusitis	3-day i.v 500 mg MP,HGG
Cogan’s syndrome												
Case 14	30/F	R	NLP	20/160	PE	(+)	35	42	(−)	(−)	Interstitial Renal Diseases, Nerve Deafness	3-day i.v 500 mg MP, plasma exchange, HGG.CCP
		L	NLP	20/80	PE							

*F*, female; *M*, male; *R*, right eye; *L*, left eye; *NLP*, no light perception; *LP*, light perception; *FC*, finger count; *HM*, hand motion; *PE*, papilledema; *OA*, optic atrophy; *Norm*, normal; *CRP*, C-reactionprotein; *ESR*, erythrocyte sedimentation rate; *ANA*, antinuclear antibody; *ANCA*, associated vasculitis; *MPO*, myeloperoxidase; *PR3*, proteinase-3; *Cr*, creatinine; *BUN*, blood urea nitrogen; *NA*, no available; (+), positive; (−),negative; *MP*, methylprednisolone; *HGG*, human gamma globulin; *CCP*, cyclophosphamide; *MTX*, methotrexate; *BIL*, bilateral; *HCQ*, hydroxychloroquine

## Literature review

Twenty-three patients with PSV-ON have been reported in the literature [9–26]. The literature review was conducted as follows: (1) Patients diagnosed with optic neuritis. (2) The optic neuropathy with characteristic changes on MRI. MRI changes include optic nerve hyperintensities in T2WI, optic nerve parenchyma/sheath enlargement and enhancement in T1WI postcontrast MRI. (3) OPN is excluded from this review. The clinical characteristics are summarized in Table 2. ON has been frequently diagnosed in AAV patients (15 cases), followed by GCA patients (6 cases). Two atypical CS patients presented ON.

**Table 2:** Clinical characteristics of patients with primary systemic vasculitis-related optic neuritis

First author (year)	Sex/Age	Eye	Optic disk	Visual Acuity	Visual Outcome	ANCA	Biopsy	Diagnosis	
Lee (1999)	F/82	R/L	Edema	R:20/400,L:NLP	R:20/400,L:NLP	(−)	TAB(+)	GCA	ESR:113 mm/1 h
	F/86	R/L	Pallor/Edema	R:FC, L:NLP	R:FC, L:NLP	(−)	TAB(+)	GCA	ESR:85 mm/1 h
	M/80	R/L	Aormal/Pallor	R:FC,L:20/20	NA	(−)	TAB(+)	GCA	ESR:95 mm/1 h, BRAO of left eye
D’Souza (2016)	M/60	L	Edema	L:FC	L:20/80	(−)	TAB(+)	GCA	ESR: 36 mm/1 h headache, diplopia, weight loss
	M/63	R/L	Edema	R/L:LP	R:FC,L: 20/320	(−)	TAB(+)	GCA	NA
Garcia-Porrua (2005)	F/82	R	Edema	R:HM	R:FC	(−)	TAB(+)	GCA	ESR:63 mm/1 h, asthenia, anorexia, headache, polymyalgia rheumatica symptoms
Salvi (2010)	F/69	R/L	Pallor	R: 1/50,L: 4/10	Improve	p-ANCA(anti-MPO) (+)	NA	AAV	Pachymeningitis
Sasaki (1995)	F/77	L	Normal	NA	NA	p-ANCA(anti-MPO) (+)	NA	AAV	Transverse myelopathy, hypertrophic pachymeningitis
Harada (1997)	M/52	R/L	NA	NA	NA	p-ANCA(anti-MPO) (+)	NA	AAV	Transverse myelopathy
Miyanaga (2021)	F/19	R/L	Normal	NA	NA	c-ANCA(anti-PR3) (+)	NA	AAV	(−)
	F/50	R/L	Normal	NA	NA	p-ANCA(anti-MPO) (+)	NA	AAV	(−)
	M/61	L	Normal	NA	NA	p-ANCA(anti-MPO) (+)	NA	AAV(GPA)	Hypertrophic Pachymeningitis,Otitismedia
Suga (2020)	M/61	R/L	Edema	R:20/20,L:NLP	R:20/20,L:20/2000	(−)	EthmoidalSinustrans(+) MaxillaryBone(+) TBLB(−)	AAV(GPA)	Fever, sinusitis, osteomyelitis, fistula of maxillary bone
Huchzermeyer (2013)	F/56	R/L	Normal	R:LP,L:20/25	R:20/25,L:20/20	c-ANCA(anti-PR3) (+)	NA	AAV(GPA)	Surgical reconstruction of the nasal septum, lacrimal duct obstruction, saddle nose deformity
Monteiro (2005)	M/32	R/L	Normal/Edema	R:FC,L:NLP	R:20/20,L:20/30	c-ANCA (+)	Nasal/Bronchial(+)	AAV(GPA)	ESR:75 mm/1 h Sinusitis, hearing loss
Fauci (1973)	F/61	L	NA	NA	NA	NA	NA	AAV(GPA)	Polyarthralgias, sinusitis, focal glornerulitis, neerolizing vasculitis, interstitial nephritis
Cutler (1956)	F/51	R/L	Normal	R/L:NLP	R/L:NLP	NA	Nasal(+)	AAV(GPA)	Anemia, nasal, sphenoidal, pulmonary, renal, joint
Moubayed (2009)	M/59	R/L	Normal	R:6/12,L:FC	R:6/7.5,L:6/21	c-ANCA (+)	TAB(-)Bronchial(+)Renal(+)	AAV(GPA)	ESR:60 mm/1 h, Polymyalgia Rheumatica
Belden (1993)	NA	R/L	Normal	R:FC,L:HM	R:FC,L:HM	ANCA (+)	Bronchial(+)	AAV(GPA)	ESR:32 mm/1 h Lung, Skin
Altaie (2005)	F/80	R	Normal	R:HM,L:6/6	R:6/9,L:6/6	p-ANCA(anti-MPO) (+)	Kidney(-)	AAV(MPA)	Asthma, sinusitis, osteoarthritis, urinary tract infection, proteinurea, haematurea, mild hearing impairment
Oshida (2004)	F/53	L	Normal	NA	NA	p-ANCA(anti-MPO) (+)	NA	AAV(MPA)	Crescentic glomerulonephritis
Bicknell (1978)	NA	NA	NA	NA	NA	NA	NA	CS	Memory loss, long thoracic neuropathy, myelopathy, phrenic neuropathy
Yamamoto (1990)	NA	NA	Normal	NA	NA	NA	NA	CS	Abducens nerve palsy, facial nerve palsy

*F*, female; *M*, male; *R*, right eye; *L*, left eye; *NLP*, no light perception; *LP*, light perception; *FC*, finger count; *HM*, hand motion; *ANCA*, anti-neutrophil cytoplasmic antibody; *MPO*, myeloperoxidase; *PR3*, proteinase-3; *TAB*, temporal artery biopsy; *TBLB*, transbronchiallung biopsy; *GCA*, giant cell arteritis; *AAV*, ANCA-associated vasculitis; *GPA*, granulomatosis with polyangiitis; *MPA*, microscopic polyangiitis; *CS*, Cogan’s syndrome; *ESR*, erythrocyte sedimentation rate; *NA*, no available; (+), positive;(−), negative

## Discussion

The ON is an exceedingly rare presentation of PSV. Even with prompt aggressive therapies, the prognosis remains poor. In all the 14 involved patients, there were no or only slight improvements in visual activity after treatments. Two of them are even worse during combination therapies.

The exact prevalence, prognosis, and pathophysiology of PSV-ON are unclear. The incidence rate of PSV is 40–54 per million cases. However, this counting may vary due to age, genetics, geographical location, and climatic changes. Clinical manifestations are variable in patients with different sizes of vascular obstruction. GCA and TAK are necrotizing vasculitides of unclear etiology, especially for medium or large vessels. They preferred to affect women. Epidemiologically, GCA affects older people, with the incidence peaking in the seventh decade. In a cohort of age over 90 years, the risk of GCA is reportedly 20 times. TAK is the most typical syndrome among Asian individuals, and 80–90% of cases are found in the 1st–3rd decades of life [27]. Several types of SVV can be associated with the serum positivity of ANCA. AAV targets capillaries, venules, and arterioles. But sometimes, middle-sized arteries can also be affected. AAV can develop at any age. Generally, AAV presents 3 major phenotypes: GPA, MPA and eosinophilic granulomatosis with polyangiitis (EGPA) [28]. ANCAs exhibit 2 distinct intracellular distribution patterns-diffuse cytoplasmic ANCAs (cANCA) directing against proteinase 3 (PR3) and perinuclear ANCAs (pANCA) against myeloperoxidase (MPO) [29]. cANCA (PR3-ANCA) is associated with GPA, while pANCA (MPO-ANCA) has been linked to MPA and EGPA. CS is a rare autoimmune multisystem inflammatory vasculitis that involves blood vessels of all sizes. CS is typically detected in young adults of any gender in their 2nd or 3rd decade of life, occasionally in middle age [30]. Although different vasculitis predilects for sizes of vessels, vascular involvement can be widespread to any size. Such associations are not necessarily exclusive and occur interchangeably. Interestingly, vasculitis peaks in particular seasons. All of our cases reported the onset during the fall and winter.

The systemic necrotizing vasculitis leads to vascular occlusion, consequently leading to poor end-organ perfusion. The brain, lungs, kidneys, and heart dysfunctions are the most common systemic presentations associated with necrotizing LVV pathology. In our research, the TAK patient (case 1) had large vessel vasculitis. He accompanied multiple organ impairments, including multiple lacunar infarcts, atypical pathogenic pneumonia, reduced left-ventricular ejection fraction, and renal dysfunction. Moreover, small vessels and retinal/choroid arteria can also impaired [31]. The interstitial or connective tissue lesion is a well-known presentation of ANCA-associated vasculitis. In this study, a higher incidence of interstitial renal/lung disease is noted among ANCA-arteritis patients (cases 10–13). GPA is a kind of ANCA-arteritis with dual-form. It presents necrotizing vasculitis as well as extravascular granulomatous inflammation. In a previous study, the ear, nose, and throat manifestations are observed in 90% of GPA patients [16]. In our study, cases 4, 9, 10 and 13 presented sinusitis and mastoiditis, representing pathologic changes of granulomatous vasculitis. MPA, another kind of ANCA-arteritis, is a systemic necrotizing vasculitis without granulomatous inflammation that distinguishes MPA from GPA. Additionally, hypertrophic pachymeningitis was present as the main feature of GPA (case 9) and MPA (case 11) in our study, as previously report [32]. Approximately 70% of CS patients have underlying vasculitis-induced systemic diseases [33] not only localized on the oculo-audio vestibular. Such as interstitial renal diseases in case 14.

The ocular manifestations of PSV have been widely reported. The tortuosity and dilatation of the retinal vessels or occlusion of the retinal artery are characteristic of retinal vasculitis. In most PSV-ON cases, localized occlusions of the retinal artery were observed at the acute phase, which eventually converted to atrophy of a sector at the later stage. GCA-associated small artery-end inflammation commonly leads to AAION [34]. TAK-associated hypertension or carotid occlusion frequently induces ocular ischemic syndromes and retinopathies. It seems ocular involvement is not caused by vasculitis directly. The anterior segment manifestations are more prominently noticed in AAV patients [2, 35, 36]. The orbital manifestations occurred in GPA specificity. A retrospective study has reported that 45% of GPA patients are accompanied by orbital infiltration due to contiguous granulomatous spreading from the paranasal sinus [37]. Typical CS is characterized by recurrent episodes of deafness and visual loss due to cochleovestibular dysfunction and interstitial keratitis, respectively. Atypical CS patients may present uveitis (37%), scleritis (23%), conjunctivitis (10%), cataract, and other ocular manifestations [30].

PSV-ON is a fatal disorder as it can lead to acute visual or central visual field loss. PSV is a critical pathogenesis for the onset of ON, although it is rarely reported. Diagnosis of PSV-ON may sometimes be delayed or misinterpreted in complex clinical manifestations of vasculitis [12, 23]. Optic nerve involvement is reportedly 0.8% in GPA and 8.0% among MPA patients [2]. Several kinds of literature have mentioned optic nerve sheath enhancement in patients with GCA [9, 10, 38, 39]. Most PSV-ON patients were positive for pANCA. Unfortunately, relevant studies have reported mixed and sometimes contradictory findings on ON.

The underlying hypothesis for the mechanism of action might be as follows. First, the penetrating pial vessels provide nutrition to the nerve parenchyma and sheath [39]. Focal vascular necrosis and hypercoagulability-induced thrombosis may cause vasculitic infarction and optic nerve ischemic damage. The second reason could be the disruption of the blood–brain barrier (BBB) permeability. Sidney et al. [40] have reported that patients with bilateral optic nerve sheath enhancement may not develop optic neuropathies. The intrinsic arteritis may not develop related optic parenchyma neuropathy. But in unrecognized or untreated vasculitis conditions, ON may onset. Third, PSV-induced autoantibodies may cross-react with neuronal cells. It is believed that in PSV-ON patients, the optic nerve is attacked by autoantibodies rather than vasculitis. The previous report confirmed the common antigens present in the inner ear and cornea in the CS patient.

There are only case series reports helping guide PSV-ON management. A prioritised treatment option is high-dose steroids at the onset. Most of these patients ultimately require a more complex regimen to induce remission and prevent PSV and ON recurrences, like a combination of biological drugs, steroids, and cyclophosphamide. The newer biological drugs, especially antagonists of interleukin-6 (IL-6) and tumor necrosis factor (TNF) might be beneficial in complicated PSV-ON cases [41]. Even with prompt therapy, GPA-associated ON often has an unfavourable prognosis. The permanent, complete or partial vision loss has been reported to be 15–20% in GCA [31]. It is hypothesised that distinctive pathology leads to divergent responses to steroids. Only half of TAK patients respond to steroids. The lack of prompt treatment may be an additional agent. Atypical CS tends to be more aggressive and has a worse prognosis. Manteiro et al. [18] have suggested that occlusive vasculitis-associated ON can be treated effectively via rapid diagnosis and aggressive anti-inflammatory therapies. However, the early-stage diagnosis of ON may sometimes be difficult due to atypical or incomplete clinical manifestations. Accordingly, optic nerve irreversible ischemic or autoimmune damage is present and without remedy methods.

## Conclusion

PSV-ON is a rare but sight-threatening disease. The autoantibodies’ attack on the optic nerve, ischemic damage, or destruction of the BBB may be potential pathogenesis. Even with prompt and aggressive clinical interventions, the prognosis remains unsatisfactory. An early diagnosis is crucial for preventing irreversible injuries to the optic nerve.

## Limitation

Our study utilized a retrospective design and was limited to a single center. There may have been some selection bias. Stringent inclusion criteria limited our sample size to 14 patients, which may have underpowered some aspects of the study. Neuromyelitis optica spectrum disorder (NMOSD) and PSV are all mult-organs involved syndromes. Optic neuritis and NMOSD-ON have overlapping clinical profiles. NMOSD-ON is not enrolled in this research. Furthermore, since all patients were identified within inpatients, there was likely selection bias toward identification.

## Data Availability

All of the data in this study were in the article. The raw data supporting the conclusions of this article will be made available by the authors, without undue reservation.
